# Nuclear RIPK3 and MLKL contribute to cytosolic necrosome formation and necroptosis

**DOI:** 10.1038/s42003-017-0007-1

**Published:** 2018-01-22

**Authors:** Kathrin Weber, Ria Roelandt, Inge Bruggeman, Yann Estornes, Peter Vandenabeele

**Affiliations:** 1 VIB Inflammation Research Center, Technologiepark 927, 9052 Zwijnaarde-Ghent, Belgium; 2 Department of Biomedical Molecular Biology, Ghent University, Technologiepark 927, 9052 Zwijnaarde-Ghent, Belgium; 3 Present Address: Université Claude Bernard Lyon 1, INSERM 1052, CNRS 5286, Centre Léon Bérard, Centre de Recherche en Cancérologie de Lyon, 69373 Lyon, France

## Abstract

Necroptotic signaling converges in the assembly of a cytosolic signaling platform, the necrosome, with the activation of its downstream effector, MLKL. RIPK1 and RIPK3, key components of the necrosome, act as signaling intermediates for the activation of MLKL. We report that RIPK3 and MLKL continuously shuttle between the nucleus and the cytoplasm, whereas RIPK1 is constitutively present in both compartments. During TNF-induced necroptosis, nuclear RIPK1 becomes ubiquitinated, after which nuclear MLKL becomes phosphorylated and oligomerized. Pharmacological inhibition of the nuclear export machinery leads to retention of RIPK3 and MLKL in the nucleus, prevents the nucleation of cytosolic RIPK3/MLKL oligomerization, and reduces cell death. Our results suggest that passage of necroptotic signaling components through the nucleus is a mechanism for regulating cytosolic necrosome formation and consequently necroptotic cell death.

## Abstract (web summary)

Kathrin Weber *et al*. report that the necrosome components RIPK3 and MLKL constitutively shuttle between the nucleus and cytoplasm. They find that increasing ratios of nuclear:cytosolic RIPK3 and MLKL prevents necrotic cell death, suggesting a mechanism by which the cell regulates necrosome formation and death.

## Introduction

Programmed necrosis (necroptosis) is a form of non-apoptotic cell death playing important roles in many inflammatory conditions and related diseases^1^. The most intensively studied model for programmed necrosis is the necroptotic pathway’s response to tumor necrosis factor (TNF). Ligand association of TNF with its cognate receptor TNF receptor (TNFR)-1 results in the formation of a membrane-associated TNFR-1 signaling complex named complex I. Within this complex, ubiquitinated receptor interacting protein 1 (RIPK1) promotes activation of the NF-κB pathway. De-ubiquitination of RIPK1 leads to the assembly of a cytosolic death complex (complex IIb), which promotes apoptotic cell death^2^ and contains active caspase-8, FAS-associated via death domain protein (FADD), RIPK1 and RIPK3. However, blocking caspase-8 activity by genetic ablation, chemical inhibitors, or viral caspase inhibitors leads to the generation of an alternative cytosolic complex IIc, the necrosome, which induces necroptotic cell death^3–5^. Mechanistically, the effector mixed-lineage kinase domain like (MLKL) is recruited to the necrosome, followed by its phosphorylation by RIPK3. This induces a conformational change in MLKL and exposes its N-terminal death effector domain (4 helical bundle domain, 4HBD). Subsequently, MLKL translocates to the plasma membrane and causes its permeabilization^6–10^.

The serine/threonine kinases RIPK1 and RIPK3 are the core components of the necroptotic signaling platform. The two proteins associate with each other through their RIP homotypic interaction motif (RHIM) domains into heteromeric RIPK1:RIPK3 complexes, and further polymerize into filamentous β-amyloid structures^11^. RIPK1 might phosphorylate RIPK3 within the necrosome, promoting the activation of RIPK3 kinase^3^, but there is no direct experimental proof yet. The RIPK3 activating function of RIPK1 can be replaced in certain circumstances by other RHIM-containing proteins, such as the TLR3/TLR4 adaptor TRIF and the DNA sensor DAI/ZBP^12–14^. In contrast to these heterodimeric activation models, recent findings revealed that chemically induced RIPK3 homo-oligomerization is sufficient to induce necroptosis^15–17^. In that situation, RIPK3 kinase activity is activated by proximity within RIPK3 oligomers. Moreover, the intracellular localization of the necrosome is also still unclear. The necrosome was described as present in detergent-insoluble fractions (NP-40/Triton X-100) as amyloid-like aggregates^11, 18^, whereas other groups have successfully immunoprecipitated complexes containing RIPK1:RIPK3 from detergent-soluble fractions^19^. Thus, the necroptotic death complex might initially form in the cytosol and subsequently migrate to the detergent-insoluble cellular compartments, such as the endoplasmic reticulum (ER), Golgi, and mitochondria-associated membranes^7, 20^. In addition, all three necroptotic key players (RIPK1, RIPK3, and MLKL) were recently found to translocate to the nucleus early in necroptosis and NLRP3 inflammasome activation^21^. However, the physiological relevance of this nuclear localization remains unknown.

Here we show that RIPK3 and MLKL are constitutive nucleo-cytoplasmic shuttling proteins. Following necroptosis induction, RIPK3 and MLKL are activated in the nucleus, and after their cooperative nuclear export, they contribute to cytosolic necrosome formation. Consequently, the export of RIPK3 and MLKL from the nucleus to the cytosol is important for necroptotic cell death.

## Results

### Nuclear RIPK3 is involved in necroptosis

**Fig. 1:**
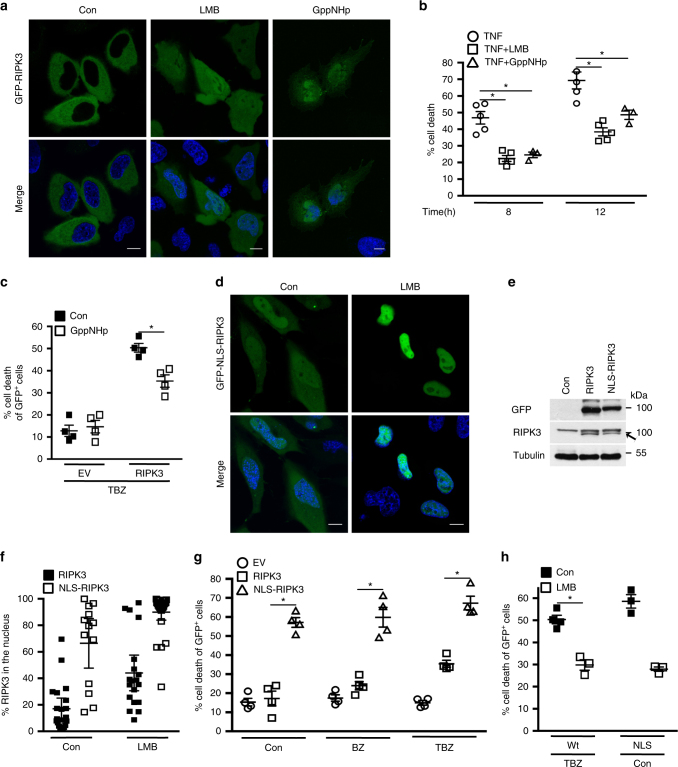
Nucleo-cytoplasmic shuttling of RIPK3 contributes to necroptosis. **a** Confocal images of single-optical sections of HeLa cells transiently transfected with GFP-RIPK3. Con: control treated; LMB: LMB treated; GppNHp: GppNHp treated. The bottom panels represent merged confocal images of GFP-tagged proteins and Hoechst (nuclear marker). Scale bars, 10 µm. **b** Cell death profile of FADD-deficient Jurkat cells pre-treated with LMB, GppNHp, or control (con) followed by TNF treatment for the indicated times. The percentage of SYTOX Green^+^ cells was analyzed and profiles are averages ± S.E.M. *n* (number of independent experiments) = 5; *p < 0.01. **c** Cell death profile of Hela cells expressing EV or GFP-RIPK3 treated with GppNHp and TBZ and analyzed by SYTOX Blue uptake in the GFP^+^ population. *n* = 4; *p < 0.01. **d** Confocal images of single-optical sections of Hela cells expressing GFP-NLS-RIPK3. Con: control treated; LMB: treated with LMB. **e** Immunoblot of expression levels of GFP-RIPK3 and GFP-NLS-RIPK3 transiently expressed in HeLa cells. Data are representative of two independent experiments. Uncropped images of immunoblots are shown in Supplementary Fig. 14. **f** Quantification of the percentage of nuclear GFP-RIPK3 (20 transfected cells of two independent experiments (*n* = 2) analyzed) and GFP-NLS-RIPK3 (28 transfected cells of one experiment (*n* = 1) analyzed) transiently expressed in HeLa cells treated or not treated with LMB. Plots indicate averages ± S.E.M. **g** Cell death profile of HeLa cells expressing empty vector (EV), GFP-RIPK3, or GFP-NLS-RIPK3 and treated with BV6 + zVAD-fmk (BZ), TNF, BV6 + zVAD-fmk (TBZ), or control (con). Cells death was analyzed by SYTOX Blue uptake in the GFP^+^ population; *n* = 4; *p < 0.01. **h** Cell death profile of HeLa cells expressing GFP-RIPK3 and treated with TBZ with or without LMB, or expressing GFP-NLS-RIPK3 and treated or untreated with LMB. Cell death was analyzed by SYTOX Blue uptake in the GFP^+^ population; *n* = 3; *p = 0.01

RIPK3 acts as a nucleo-cytoplasmic shuttling protein^22^. We confirmed that in the steady state, GFP-RIPK3 was diffusely present predominantly in the cytoplasm, and that inhibition of nuclear export by Leptomycin B (LMB) led to retention of 44 ± 3.4% of total GFP-RIPK3 in the nucleus (Fig. 1a, f). Thus, RIPK3 continuously shuttles between the cytoplasm and the nucleus.

As RIPK3 is a key mediator of necroptotic cell death, we reasoned that nuclear-cytoplasmic shuttling of RIPK3 could affect necroptosis. When FADD-deficient Jurkat cells are treated with TNF, they undergo RIPK3-dependent necroptosis^3, 23^. In agreement, we showed that 8 h of TNF treatment resulted in the death of FADD-deficient Jurkat cells (46 ± 3.7%). This cell death was inhibited by an inhibitor of RIPK1 kinase activity (Nec-1), but not by a general caspase inhibitor (zVAD-fmk) (Supplementary Fig. 1, Fig. 1b). Inhibition of nuclear export by LMB reduced necroptosis induction from 46 ± 3.7% to 22 ± 1.7% (at 8 h), and this inhibitory effect was maintained over time (from 69 ± 5.1% to 38 ± 2.4% at 12 h) (Fig. 1b). We validated these results by LMB treatment in another classical necroptotic model system in which mouse embryonic fibroblasts (MEF) are treated with a combination of TNF, Tak1 inhibitor (Taki) and zVAD-fmk^24, 25^. Addition of LMB reduced necroptosis in MEF cells to an extent resembling that observed in FADD-deficient Jurkat cells (Fig. 1b, Supplementary Fig. 2). To validate the role of nuclear compartmentalization in necroptotic signaling, we inhibited nuclear import by a non-hydrolyzable GTP analog (GppNHp). Nuclear import requires the hydrolysis of GTP by Ran, which recycles the import carriers (importins) in the cytosol in order to initiate new rounds of cargo import^26–28^. GppNHp reduced not only TNF-induced necroptosis in FADD-deficient Jurkat cells to 24 ± 1.7% at 8 h (Fig. 1b), but also necroptosis induced by TNF, BV6 smac mimetic (which depletes cIAPs^29^), and zVAD-fmk (TBZ) in GFP-RIPK3-expressing HeLa cells from 50 ± 1.9% to 35 ± 2.8% (Fig. 1c). However, GppNHp did not inhibit nuclear import of GFP-RIPK3, which accumulated in the nucleus, recapitulating the intracellular distribution observed in the presence of LMB (Fig. 1a). Consequently, the nuclear import mechanism of GFP-RIPK3 is distinct from the classical Ran-GTP-dependent nuclear import mechanism. It is likely that GppNHp inhibits the import of a protein required for RIPK3 nuclear export.

If reduction of necroptosis by LMB/GppNHp is evoked by interference with the nuclear passage of RIPK3, then altering nucleo-cytoplasmic shuttling of RIPK3 should affect necroptosis induction. A commonly used strategy to modulate nucleo-cytoplasmic shuttling is to inactivate intrinsic nuclear export signal (NES) or NLS sequences by point mutation of core residues. However, sequence analysis did not reveal any conventional NLS or NES within RIPK3. The absence of a conventional NLS in RIPK3 is in agreement with the unaltered nuclear import of RIPK3 in the presence of GppNHp (Fig. 1a). Furthermore, the existence of an unconventional C-terminal NLS sequence (aa 442–472) could not be experimentally confirmed^30, 31^. In agreement with an earlier report^30^, we confirmed that the kinase domain of RIPK3 (aa 1–292) is imported into the nucleus, demonstrating the dispensable role of the RIPK3 C-terminal region in nuclear import (Supplementary Fig. 3). In order to increase nuclear-cytoplasmic shuttling of RIPK3 we fused the NLS sequence of Simian Vacuolating Virus (SV)40 large T antigen, which serves as a consensus NLS sequence, to the N-terminus of RIPK3 (NLS-RIPK3). Thus, NLS-RIPK3 is subjected to the classical Ran-GTP nuclear import mechanism in order to bypass any alternative TBZ-dependent import mechanisms. When GFP-NLS-RIPK3 was expressed in HeLa cells, in which endogenous RIPK3 expression is silenced by promotor methylation^32^, it was detected in a diffuse pattern throughout the cell, and 66 ± 8.6% of total NLS–RIPK3 was in the nucleus (Fig. 1d, f). In the presence of LMB, 90 ± 3% of the entire cellular pool of NLS–RIPK3 accumulated in the nucleus. These results indicate that NLS-RIPK3 recapitulated nucleo-cytoplasmic shuttling of wild-type RIPK3 but with a more efficient import rate. Thus, NLS–RIPK3 can be used to analyze whether increased nucleo-cytoplasmic shuttling, and hence nuclear passage of RIPK3, correlates with sensitization to necroptosis. Although RIPK3 and NLS-RIPK3 were expressed at comparable levels, only NLS-RIPK3 exerted pro-death activity (57 ± 2.6% cell death) at steady state (Fig. 1e, g). Treatment with TBZ enhanced cell death from 15 ± 2% to 35 ± 1.8% when HeLa cells expressed wild-type RIPK3 but only marginally increased NLS-RIPK3-mediated cell death, from 57 ± 2.5% to 67 ± 3.6%. Treatment of wild-type RIPK3-expressing HeLa cells with LMB reduced both steady-state NLS–RIPK3-induced cell death (from 58 ± 3.0% to 28 ± 1.0%) and TBZ-induced necroptosis (from 50 ± 2.0% to 30 ± 2.8%) (Fig. 1h). Taken together, these results suggest that passage of RIPK3 through the nuclear compartment contributes to TNF-induced necroptosis.

### Nuclear RIPK1 is ubiquitinated during necroptosis

**Fig. 2:**
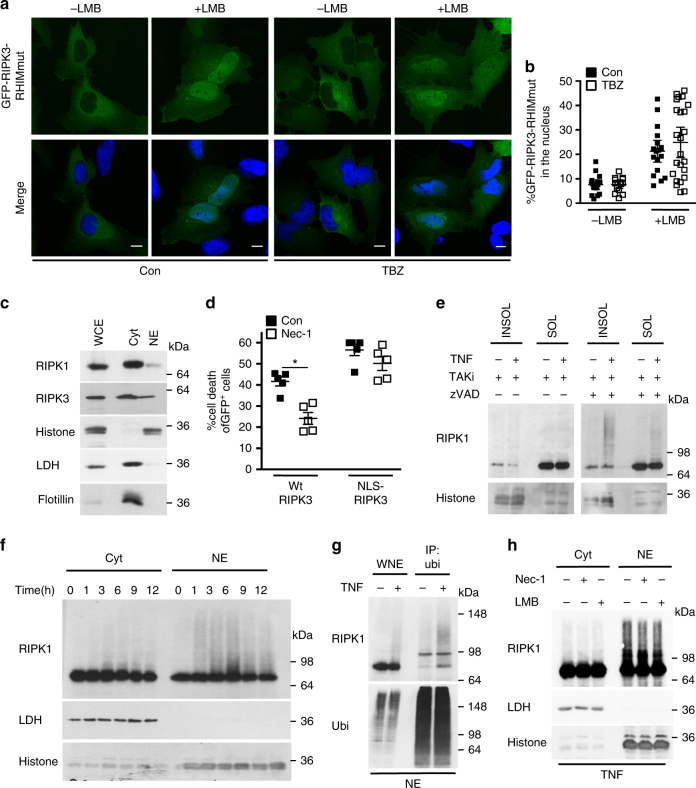
Nuclear RIPK1 is ubiquitinated during necroptosis. **a** Confocal images of single-optical sections of HeLa cells expressing GFP-RIPK3 RHIMmut treated with control (con) or TNF, BV6 and zVAD-fmk (TBZ), with or without LMB. Scale bars: 10 µm. **b** Quantification of the percentage of nuclear GFP-RIPK3-RHIMmut transiently expressed in HeLa cells and treated with control (con) or with TBZ, with or without LMB. 18–25 transfected cells of a representative experiment of three independent experiments were analyzed. Plots indicate averages ± S.E.M. **c** Immunoblot of intracellular distribution of RIPK1 and RIPK3 in FADD-deficient Jurkat cells comparing actual protein levels. Lactate dehydrogenase (LDH): cytosolic marker; Histone: nucleus; Flotillin: lipid rafts. Cyt: cytosolic fraction; NE: nucleus-enriched fraction; WCE: whole-cell extract (10%). **d** Cell death profile of HeLa cell expressing either GFP-RIPK3 or GFP-NLS-RIPK3 treated with TBZ with or without Nec-1 analyzed by SYTOX Blue uptake in the GFP+ population. *n* = 5; *p < 0.01. **e** Immunoblots of RIPK1 in the 1% NP-40-soluble and -insoluble fractions of wild-type MEF cells treated with TT (TNF and TAKi) or TTZ for 4 h. **f** Immunoblot of RIPK1 in cytoplasmic and nuclear fractions isolated from FADD-deficient Jurkat cells treated for the indicated times with TNF. RIPK1 levels were equalized between nuclear and cytosolic fractions by loading four times more of the nucleus-enriched fraction. **g** Immunoblot of RIP1 in anti-ubiquitin immunoprecipitation of NE of FADD-deficient Jurkat cells treated with TNF for 3 h. WNE: whole-nuclear extract. **h** Immunoblot of the relative amounts of cytoplasmic and nuclear RIPK1 of FADD-deficient Jurkat cells pre-treated with Nec-1 or LMB and then with TNF (3 h). All immunoblots are representative of two or three independent experiments. Uncropped images of immunoblots are shown in Supplementary Fig. 15

Following necroptosis induction, initial cytosolic complexes containing RIPK1:RIPK3 translocate to different cellular compartments, such as mitochondria associated membranes, where they exert their function^7, 20^. We hypothesized that RIPK3 could be imported into the nucleus as a pre-assembled RIPK1:RIPK3 complex. If that was true, interference with the RIPK1:RIPK3 interaction interface would prevent nuclear import of RIPK3. The RHIM motif of RIPK3 represents the interface for association with RIPK1, and tetra alanine substitution of core residues within the RHIM domain of RIPK3 abolishes its interaction with RIPK1^3, 33^. Addition of LMB led to nuclear retention of 42 ± 4.2% of this tetra alanine RIPK3 mutant (RIPK3-RHIMmut) in control-treated conditions and 49 ± 6% in TBZ-treated conditions (Fig. 2a, b). Apparently, the nucleo-cytoplasmic shuttling of RIPK3-RHIMmut was not altered compared to wild-type RIPK3, as LMB led to nuclear retention of 44 ± 4.2% of RIPK3-RHIMmut and to 44 ± 3.4% of wild-type RIPK3 (Fig. 1a, f). This indicates that the RHIM domain of RIPK3 (and hence homotypic interaction with RIPK1) did not mediate the nuclear import of RIPK3 following TNF-induced necroptosis.

RIPK1 has been reported to localize in the nucleus at steady state^34^. In line with this, we found RIPK1 in nuclear fractions of control FADD-deficient Jurkat cells (Fig. 2c), suggesting a possible role for nuclear RIPK1 in necroptotic signaling. The kinase activity of RIPK1 is required for TNF-induced necroptosis to promote association with and activation of RIPK3, whereas Nec-1, an allosteric inhibitor of RIPK1 kinase activity, efficiently blocks necroptosis^35^. We found that Nec-1 restored cell viability in RIPK3-expressing HeLa cells treated with TBZ, but it did not affect NLS–RIPK3-induced cell death (Fig. 2d), showing that in the latter case NLS-RIPK3-mediated cell death does not require RIPK1 kinase activity. This suggests that mechanisms other than RIPK1-dependent activation may be involved in NLS–RIPK3-induced necroptosis, but not in TBZ-induced necroptosis involving wild-type RIPK3.

RIPK1 has been reported to be regulated by ubiquitination within the necroptotic signaling complex and consequently in the NP-40-insoluble fraction^36, 37^. In agreement with these reports, we observed modified RIPK1 as slower migrating forms on an immunoblot of MEF cells induced to die by necroptosis by (TTZ) TNF, Tak1 inhibitor (Taki) and zVAD-fmk treatment (Fig. 2e). Importantly, modified RIPK1 was found almost exclusively in the NP-40-insoluble fraction. As nuclei are largely insoluble in non-ionic detergents like NP-40, these results imply that RIPK1 might be ubiquitinated in the nucleus. Indeed, when RIPK1 levels were equalized between nuclear and cytosolic fractions by loading four times more of the nucleus-enriched fraction, we observed similar slower migrating forms of RIPK1 in the nucleus-enriched fractions of necroptotic FADD-deficient Jurkat cells (Fig. 2f). This nuclear RIPK1 ubiquitination was not reduced by smac mimetic, indicating that cIAPs are dispensable for this post-translational modification in the nucleus (Supplementary Fig. 4a).

To determine the type of modification on nuclear RIPK1, we performed anti-ubiquitin immunoprecipitation on nuclear fractions of TNF-treated FADD-deficient Jurkat cells. RIPK1 was recovered only after necroptosis induction, indicating its ubiquitination (Fig. 2g). Supporting a non-degradative function of nuclear RIPK1 ubiquitin chains, proteasome inhibition by MG132 had no effect on the protein levels of nuclear RIPK1 (Supplementary Fig. 4b). Although inhibition of RIPK1 kinase activity by Nec-1 has been reported to reduce RIPK1 ubiquitination^36^, we did not observe any dependency of RIPK1 kinase activity on its ubiquitination in FADD-deficient Jurkat cells (Fig. 2h). Furthermore, the presence of Nec-1 did not affect RIPK1 levels in the nucleus (Fig. 2h). This observation contradicts the reported necessity of RIPK1 kinase activity for its nuclear import during necroptosis^21^. Addition of LMB had no effect on nuclear RIPK1 levels, indicating that RIPK1 is not exported from the nucleus independently of CRM1. Altogether, these results indicate that RIPK1 is ubiquitinated in the nucleus during necroptosis, but this ubiquitination is not affected by LMB.

### RIPK3 is active in the nucleus

**Fig. 3:**
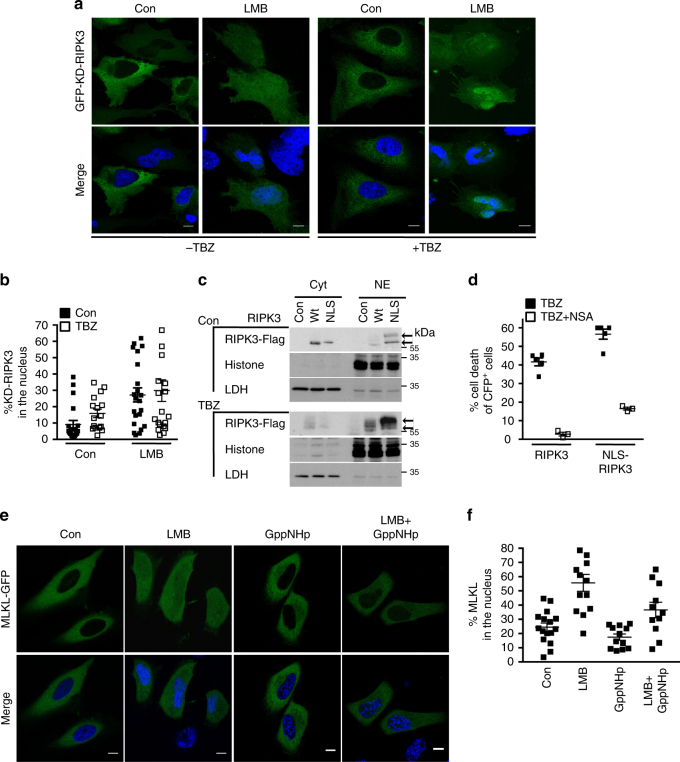
RIPK3 kinase is active in the nucleus. **a** Confocal images of single-optical sections of Hela cells transiently expressing kinase-dead KD-RIPK3. Scale bars: 10 µm. **b** Quantification of the percentage of nuclear GFP-RIPK3 D160N transiently expressed in HeLa cells treated with TBZ with or without LMB or control-treated. 14–20 transfected cells of a representative experiment of two independent experiments were analyzed. Plots indicate averages ± S.E.M. **c** Immunoblot of Flag-RIPK3 in nuclear and cytosolic fractions of TBZ or control treated HeLa cells expressing EV, RIPK3 or NLS-RIPK3. Immunoblots are representative of two independent experiments. Uncropped images of Western blots are shown in Supplementary Fig. 16. **d** Cell death profile of HeLa cells transiently expressing CFP-RIPK3 or CFP-NLS-RIPK3 pre-treated with NSA or control (con) followed by treatment with TBZ; SYTOX Red uptake in the CFP^+^ population was analyzed; *n* = 3. **e** Confocal images of single-optical sections of HeLa cells transiently transfected with MLKL-GFP. Left panel: con: control treated; LMB: treated with LMB; GppNHp: treated with GppNHp; LMB + GppNHp: treated with LMB and GppNHp. Scale bars: 10 µm. **f** Quantification of the percentage of nuclear MLKL-GFP transiently expressed in HeLa cells treated with control (con), LMB, GppNHp, or LMB + GppNHp. 13–24 transfected cells were analyzed of one or two independent experiments (*n* = 1 (GppNHp treated); *n* = 2 (con, LMB and GppNHp + LMB treated). Plots indicate averages ± S.E.M

It has been proposed that ubiquitination of RIPK1 within the RIPK1:RIPK3 necrosome during TNF signaling influences the phosphorylation and hence the activation of RIPK3^36, 37^. As we observed ubiquitination of RIPK1 in the nucleus (Fig. 2f), we hypothesized that a RIPK3-activating complex is formed in the nucleus. To test this hypothesis, we first examined whether the kinase activity of RIPK3 is required for its nuclear import. Kinase-dead RIPK3 (D160N) exhibited a nucleo-cytoplasmic shuttling capacity resembling that of wild-type RIPK3, as LMB led to nuclear retention of 27 ± 4.3% of kinase-dead RIPK3 compared to 44 ± 6.4% of wild-type RIPK3 (Figs. 3a, b, 1f). In addition, kinase-dead RIPK3 apparently failed to associate with its substrate, MLKL, indicating that the nuclear import of RIPK3 is independent of its binding to MLKL (Supplementary Fig. 5). Furthermore, wild-type RIPK3 was detected as a double band only in nucleus-enriched fractions following TBZ treatment. Also, modified NLS–RIPK3 was present only in nuclear extracts at steady state and after TBZ treatment (Fig. 3c). Lambda phosphatase treatment confirmed that the observed mobility shift of NLS–RIPK3 and wild-type RIPK3 in the nuclear fractions represented phosphorylated (i.e., active) RIPK3 (Supplementary Fig. 6). Together, these results point to activation of RIPK3 in the nucleus and prompted us to study the involvement of MLKL in nuclear necroptotic signaling.

### MLKL is a constitutive nucleo-cytoplasmic shuttling protein

The activation of human MLKL can be inhibited by necrosulfonamide (NSA), which covalently bonds to MLKL Cys 86, preventing its oligomerization but not its phosphorylation^19^. As MLKL is the endpoint of necroptotic signaling, NSA should inhibit cell death induced by wild-type RIPK3 or NLS-RIPK3. Indeed, NSA reduced to a similar extent the cell death induced by NLS–RIPK3 (from 41 ± 2.1% to 3 ± 1.4%) or by TBZ treatment of wild-type RIPK3-expressing cells (from 56 ± 2.7% to 16 ± 0.8%) (Fig. 3d).

MLKL contains a NLS sequence that is occluded in the native conformation, and so it is found only in the cytoplasm in the steady state. Following necroptosis induction, phosphorylation of MLKL by RIPK3 triggers a conformational change that exposes the NLS, facilitating MLKL import into the nucleus^21^. We showed that inhibiting the nuclear export machinery with LMB led to retention of 55 ± 5.6% of total cellular MLKL in the nucleus at steady state (Fig. 3e, f). Thus, like RIPK3, MLKL is a constitutive nucleo-cytoplasmic shuttling protein that is exported from the nucleus in a LMB-sensitive way. In contrast to RIPK3, MLKL is apparently imported by the classical Ran-GTP/importin-dependent pathway, because inhibition of nuclear import by GppNHp reduced LMB-induced nuclear accumulation of MLKL to 36 ± 5.5% (Fig. 3e, f). As GppNHp did not affect RIPK3 import (Fig. 1a) and the constitutive association of a distinct cellular pool of RIPK3 with MLKL (Supplementary Fig. 5; ref. ^19^), we speculate that MLKL could be the GppNHp-sensitive transporting protein that allows the exit of RIPK3 from the nucleus.

To gain further insight into whether nuclear MLKL affects necroptosis, we employed the experimental approach we had used for RIPK3 and fused the canonical NLS sequence of SV40 to the C-terminus (kinase domain) of MLKL. Despite increased protein levels, ectopic expression of wild-type or MLKL-NLS in addition to endogenous MLKL did not enhance necroptosis compared to control-transfected cells (Supplementary Fig. 7a, b; ref. ^19^). Thus, MLKL expression levels did not correlate with necroptosis induction. This observation is in line with the accumulation of high levels of MLKL without cell death induction as long as the ESCRT system is operating^38, 39^.

### MLKL is active in the nucleus

**Fig. 4:**
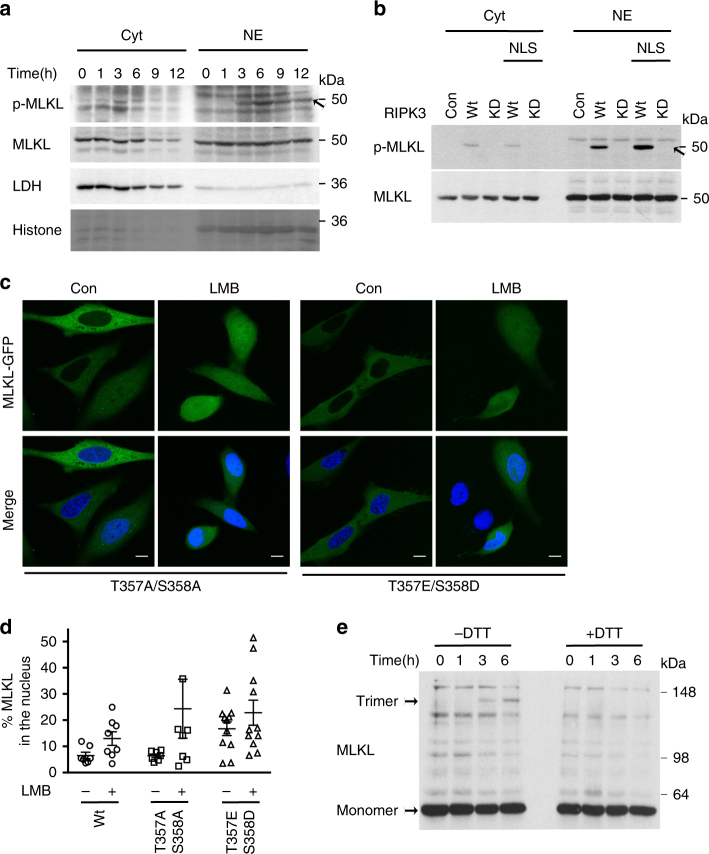
MLKL is active in the nucleus. **a** Immunoblot of phospho-MLKL (p-MLKL) showing comparison of MLKL levels in nuclear and cytosolic fractions of TNF-treated FADD-deficient Jurkat cells. MLKL levels were equalized between nuclear and cytosolic fractions by loading four times more of the nucleus-enriched fraction. **b** Immunoblot of p-MLKL showing comparison of MLKL levels in nucleus-enriched and cytosolic fractions of HeLa cells expressing untagged-RIPK3, NLS-RIPK3, or the corresponding kinase-dead mutants. **c** Confocal images of single-optical sections of HeLa cells transiently expressing MLKL-GFP, phosphodeficient (T357A/S358A) MLKL-GFP or phosphomimetic (T357E/S358D) MLKL-GFP, and Flag-RIPK3. Scale bars: 10 µm. **d** Quantification of the percentage of nuclear MLKL-GFP, phosphodeficient (T357A/S358A) MLKL-GFP or phosphomimetic (T357E/S358D) MLKL-GFP transiently transfected in HeLa cells, which also co-expressed Flag-RIPK3 and were treated or untreated with LMB. For wild-type MLKL 8 (LMB untreated) and 9 (LMB treated) transfected cells were analyzed; for phosphodeficient MLKL 12 (for both LMB untreated or treated) transfected cells and for phosphomimetic MLKL 8 (LMB untreated) and 9 (LMB treated) transfected cells were analyzed. *n* = 1. Plots indicate averages ± S.E.M. **e** Immunoblot of MLKL under non-reducing and reducing conditions of nucleus-enriched fraction from FADD-deficient Jurkat cells treated with TNF for the indicated times and the redox catalyst copper(II)(1,10-phenanthroline)3 (CuPhe). All immunoblots are representative of two or three independent experiments. Uncropped images of Western blots are shown in Supplementary Fig. 17

Our data suggest that RIPK3 kinase is phosphorylated, i.e., active in the nucleus. Therefore, MLKL as a substrate of RIPK3 could also be phosphorylated in the nucleus. Indeed, we detected phosphorylated MLKL (phospho-MLKL) in the nucleus-enriched fractions of FADD-deficient Jurkat cells after 3 h of TNF treatment when MLKL levels in the cytosolic and nucleus-enriched fractions were equalized by loading four times more nucleus-enriched fraction (Fig. 4a). At this time point, nuclear RIPK1 was ubiquitinated but cells were not positive for SYTOX Green (Fig. 2f, h; Supplementary Fig. 1). After 6 h of TNF treatment, SYTOX green-positive cells were detected (Supplementary Fig. 1). To provide further evidence that RIPK3 may phosphorylate MLKL in the nucleus, kinase-dead wild type or the corresponding NLS-tagged RIPK3 variant were expressed in HeLa cells and the phosphorylation status of endogenous MLKL was determined (Fig. 4b). Phospho-MLKL was observed in nuclear extracts only when HeLa cells expressed wild type or NLS-RIPK3, but not the corresponding kinase-dead mutants (Fig. 4b). Furthermore, the intensity of the p-MLKL band was increased when cells expressed NLS-RIPK3 compared to wild-type RIPK3 (Fig. 4b), likely due to enhancement of the pro-death activity of NLS-RIPK3 (Fig. 1g). These results confirm that RIPK3 phosphorylates MLKL in the nucleus.

However, it was previously suggested that phosphorylation of MLKL is needed for nuclear import^21^. To investigate if initial phosphorylation of MLKL in the cytosol dictates its import into the nucleus, we analyzed the nucleo-cytoplasmic shuttling of phospho-deficient and phospho-mimetic MLKL mutants (T357A/S358A and T357E/S358D, respectively). In the absence of LMB, phospho-deficient MLKL was retained in the nucleus more than wild-type MLKL or phospho-mimetic MLKL, indicating that phosphorylation of MLKL is required for its export rather than its import (Fig. 4c, d). Moreover, when LMB was co-expressed with wild-type RIPK3, it led to similar nuclear retentions: 22 ± 4.8% of phospho-mimetic MLKL, 24 ± 11.2% of phospho-deficient MLKL, and 17 ± 2.6% of wild-type MLKL (Fig. 4c). Hence, phosphorylation of MLKL and its associated conformational change might not be required for its nuclear import.

It has been reported that following its phosphorylation, MLKL assembles into disulfide-linked, SDS-stable oligomers^6, 40^. Following TNF treatment, a band running at the height of putative trimeric MLKL was observed in nuclear extracts (Fig. 4e). The integrity of these disulfide-linked lower-order MLKL oligomers (e.g., trimers or tetramers) was ensured by their absence under reducing conditions. This result is in agreement with the described sequence of events during MLKL activation^19^, namely, the detection of p-MLKL associated with the appearance of lower-order MLKL oligomers (Fig. 4a). Together, our results are consistent with the presence of phosphorylated RIPK3 and the phosphorylation and oligomerization of MLKL in the nucleus during TNF-induced necroptosis.

### Nuclear active RIPK3 and MLKL oligomerize in the cytosol

**Fig. 5:**
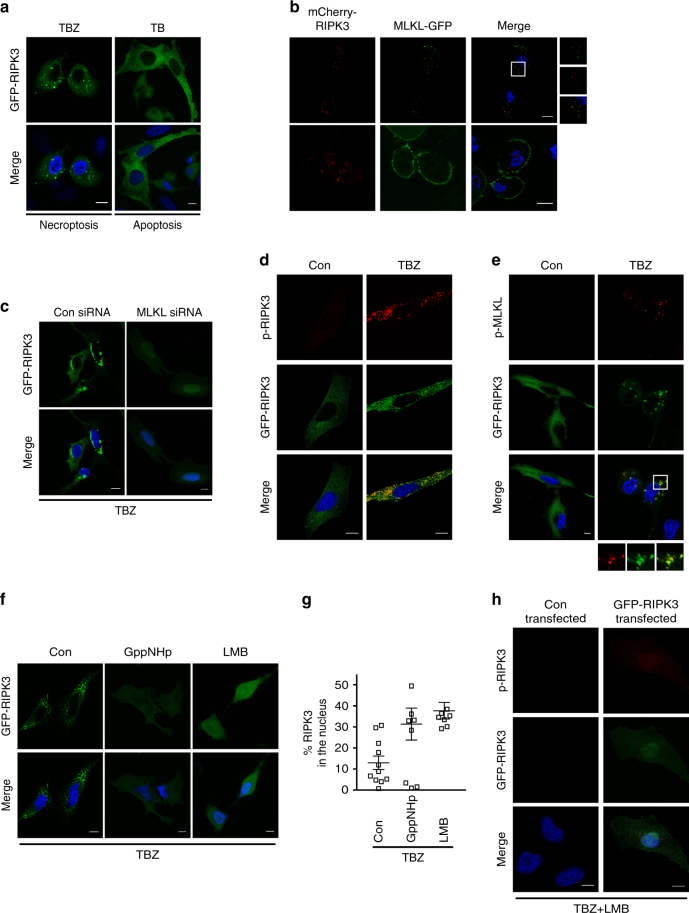
LMB and GppNHp prevent oligomerization of cytosolic RIPK3:MLKL. **a** Confocal imaging of single-optical sections of HeLa cells transiently expressing GFP-RIPK3 treated with TBZ (TNF, BV6, and zVAD.fmk) or TB (TNF and BV6). **b** Confocal imaging of single-optical sections of HeLa cells transiently expressing mCherry-RIPK3 and MLKL-GFP. Shown are merged images of mCherry-RIPK3, MLKL-GFP and Hoechst; inset is magnified. **c** Confocal images of single-optical sections of HeLa cells transiently expressing GFP-RIPK3 and transfected with control (con) siRNA or MLKL siRNA and treated with TBZ. Representative images of two independent experiments are shown. **d** Confocal images of single-optical sections of HeLa cells transiently expressing GFP-RIPK3, TBZ treated or untreated followed by anti-human p-RIPK3 immunostaining. Representative images of two independent experiments are shown. **e** Confocal images of single-optical sections of HeLa cells transiently expressing GFP-RIPK3, TBZ treated or untreated followed by anti-human p-MLKL immunostaining. Inset is magnified. Representative images of three independent experiments are shown. **f** Confocal images of single-optical sections of HeLa cells transiently expressing GFP-RIPK3, treated with TBZ, TBZ + GppNHp or TBZ + LMB. Representative images of two independent experiments are shown. **g** Quantification of the percentage of nuclear GFP-RIPK3 transiently expressed in HeLa cells treated with TBZ, TBZ + GppNHp, and TBZ + LMB (10–40 transfected cells were analyzed of *n* = 2). Plots indicate averages ± S.E.M. **h** Confocal images of single-optical sections of HeLa cells transiently transfected with control vector or GFP-RIPK3, treated with TBZ + LMB, and immunostained with an anti-human p-RIPK3 antibody. Representative images of two independent experiments are shown. Scale bars in all images: 10 µm

Following necroptosis induction, RIPK3 forms amyloid-like platforms, which are seen as cytosolic dots and are associated with necrosome formation^11, 19, 41^. We confirmed that overexpressed GFP-RIPK3 formed discrete dots in the cytosol of HeLa cells treated with TBZ^19^ (Fig. 5a); the spots were not evident under apoptotic conditions (TNF and BV6). Considering the appearance of nuclear, lower order MLKL oligomers during necroptosis (Fig. 4e), we hypothesized that MLKL might exit the nucleus complexed with RIPK3 to nucleate the formation of higher order oligomers in the cytosol. If so, the observed cytosolic GFP-RIPK3 dots would also contain MLKL. Accordingly, after co-expression of MLKL-GFP and mCherry-RIPK3 at toxic levels, both RIPK3 and MLKL localized in cytosolic dot-like structures (Fig. 5b). Besides this particular fluorescence pattern, cells without obvious cytosolic MLKL-GFP dots were also evident. In these cells, MLKL-GFP was present in a ring-like pattern at the cell periphery while mCherry-RIPK3 was still detectable as cytosolic dots (Fig. 5b). This staining could represent MLKL recruited to the plasma membrane to participate in membrane permeabilization and hence, would characterize cells further downstream in the necroptotic signaling pathway^6^. Further evidence for the requirement of MLKL in the formation of cytosolic GFP-RIPK3 dots was provided by the absence of such dots when MLKL was knocked down (Fig. 5c). Instead, GFP-RIPK3 was present as a diffuse pattern in the cytosol and the nucleus, indicating impairment of its export from the nucleus. Importantly, expression levels of RIPK3 were comparable between control siRNA and MLKL siRNA transfections (Supplementary Fig. 8). Thus, MLKL appears to mediate the nuclear export of RIPK3 and to promote cytosolic formation of higher order RIPK3 platforms. Since our data suggest that phospho-RIPK3 and phospho-MLKL are formed in the nucleus and subsequently exported cooperatively to assemble into higher order complexes in the cytosol, cytosolic RIPK3:MLKL dots should contain phospho-RIPK3 and phospho-MLKL. Indeed, immunostainings of phospho-RIPK3 and endogenous phospho-MLKL in HeLa cells expressing GFP-RIPK3 and treated with TBZ revealed cytosolic dots co-localized with GFP-RIPK3 cytosolic dots (Fig. 5d, e). To validate these results on endogenous levels in MEF cells, we tested the available anti-mouse phospho-RIPK3 and anti-mouse phospho-MLKL antibodies on these cells (Supplementary Fig. 9). However, though the immunostaining patterns were likely specific, they were not sufficiently over the threshold of background fluorescence, preventing interpretation of the results.

Inhibition of nuclear export machinery by LMB led to an increase in nuclear retention from 13 ± 3.2% to 37 ± 3.9% of total cellular GFP-RIPK3 (Fig. 5g). Likewise, inhibition of nuclear import by GppNHp led to nuclear retention of 31 ± 7.6% of total cellular GFP-RIPK3. In both conditions, TBZ-induced cytosolic GFP-RIPK3 dots were not induced (Fig. 5f, g). Moreover, no cytosolic p-RIPK3 dots were seen when nuclear export was inhibited by LMB. Instead, a diffuse human phospho-RIPK3 immunostaining was visible in the nucleus (Fig. 5h). As GppNHp did not inhibit nuclear import of RIPK3 but affected its export, we speculate that GppNHp leads to retention of phospho-RIPK3 in the nucleus. Consequently, GppNHp treatment would result in retention of phospho-RIPK3 in the nucleus, giving a phospho-RIPK3 immunostaining comparable to the one observed following LMB treatment. Analysis of the intracellular distribution of endogenous human phospho-MLKL in the presence of TBZ + LMB showed that no cytosolic p-MLKL dots were present. However, a nuclear diffuse human phospho-MLKL immunostaining was not detected: the immunofluorescence signal might have been below the detection limit and not over the threshold of background fluorescence (Supplementary Fig. 10). These results show that nuclear export is important for the formation of phospho-MLKL dots in the cytosol. Taken together, these data strongly suggest that RIPK3 phosphorylates MLKL in the nucleus and then they are cooperatively exported to the cytosol, where phospho-RIPK3 and phospho-MLKL form higher order functional platforms in the cytosol.

## Discussion

**Fig. 6:**
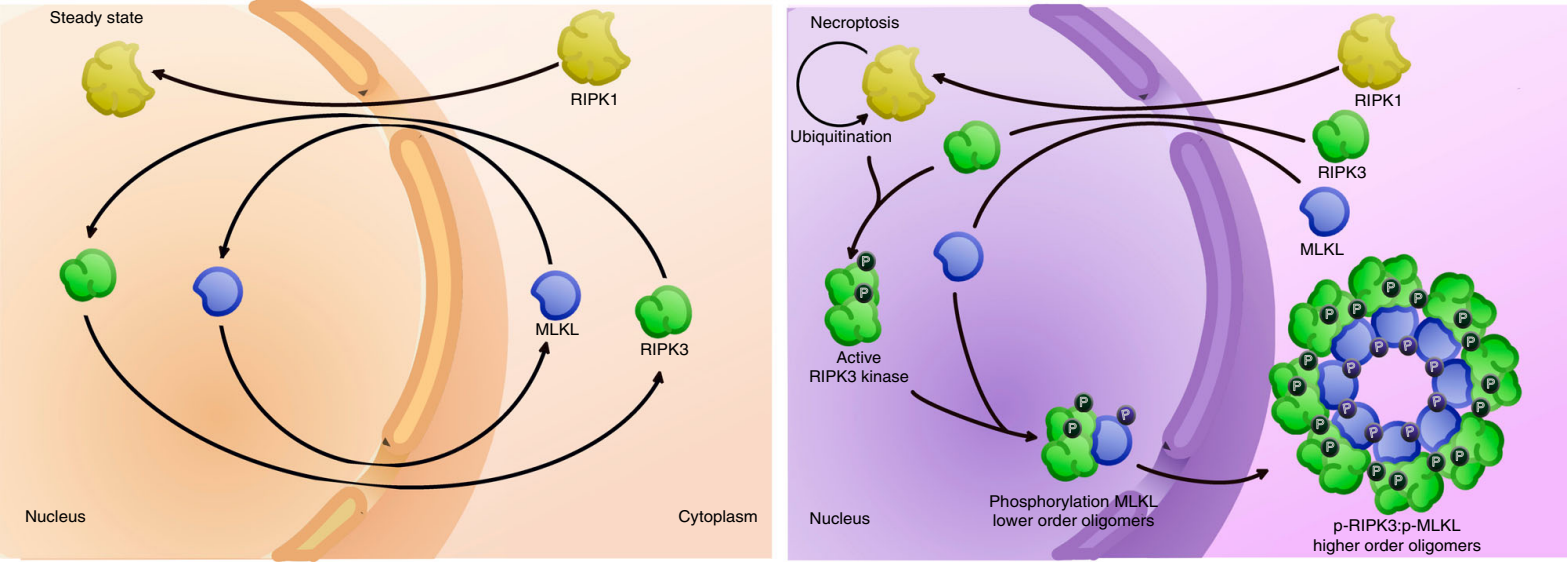
Nuclear RIPK3 and MLKL contribute to cytosolic RIPK3:MLKL oligomerization during necroptosis. Hypothetical model of the nuclear events following necroptosis induction. Nuclear RIPK1 is ubiquitinated to provide a scaffold for nuclear RIPK3 kinase activation. After its phosphorylation, RIPK3 associates with MLKL, leading to phosphorylation of MLKL. p-RIPK3 and p-MLKL are then cooperatively exported from the nucleus to contribute to cytosolic necrosome formation and consequently to cell death

The effector molecule of necroptosis, MLKL, is activated on a RIPK3-containing signaling platform, the cytosolic necrosome. This has been visualized by confocal imaging and was described as the formation of cytosolic RIPK3 dots^11, 19, 41^. Here we show that formation of cytosolic RIPK3 dots apparently depends on the export of RIPK3 and MLKL from the nucleus (Fig. 6). In the nucleus, RIPK3 and MLKL are phosphorylated and oligomerized. Thus, the joint nuclear export of phospho-RIPK3 and phospho-MLKL to the cytosol is necessary for nucleation of the necrosome in the cytosol and subsequent cell death. However, the extent to which nuclear passage is important for necroptosis induction is unknown. Our finding that LMB and GppNHp reduce necroptosis only by half (Fig. 1) may reflect the redundancy and adaptability of biological systems, which produce partial effects when one regulator is blocked. Another conclusion might be that nuclear passage and activation of RIPK3 and MLKL is a partial amplifying mechanism enhancing and accelerating necroptosis execution.

Following TNF-mediated induction of necroptosis, RIPK1 was reported to be ubiquitinated within the RIPK1–RIPK3 necrosome^35, 36^. We detected ubiquitinated RIPK1 in the nucleus (Fig. 2), suggesting that a protein complex containing RIPK1–RIPK3 formed in the nucleus promotes the activation of RIPK3 kinase activity in the nucleus. In support of this hypothesis, we showed that the kinase activity of RIPK3 was not required for its nuclear import (Fig. 3a) and that RIPK3 is phosphorylated and hence activated in the nucleus (Fig. 3c). Following its activation, RIPK3 phosphorylates MLKL, which leads to its oligomerization. In accordance with the nuclear localization of active RIPK3, we also detected its prototype substrate, MLKL, and the consequent formation of MLKL-containing lower-order oligomers in the nucleus (Fig. 4a, e). Like RIPK3, MLKL phosphorylation is not necessary for its import (Fig. 4c), indicating that these phosphorylation-dependent activation events happen in the nucleus.

Kinases release their substrates after phosphorylating them. However, in RIPK3-mediated phosphorylation of MLKL, a pronounced conformational change induced in MLKL results in the release of its 4HBD^9^ and in helix α6 mediated oligomerization^42^. These structural changes may allow stabilized interaction between the kinase domain of RIPK3 and the pseudokinase domain of MLKL, which leads to formation of a phospho-RIPK3:phospho-MLKL complex^43^. Such a complex might be exported from the nucleus in a cooperative manner (Fig. 3e, 5c, f), resulting in further nucleation of cytosolic necrosome complexes by RIPK3 amyloid-like platform formation^43^. In that scenario, the nuclear export of phospho-RIPK3 is needed to promote cytosolic phospho-RIPK3:phospho-MLKL oligomerization (Fig. 5h).

The assembly of higher order signaling platforms, such as the necrosome is commonly seeded by polymerization of pre-formed subunits (protomers). Thus, the observed nuclear lower-order MLKL oligomers (Fig. 4e) may represent a p-RIPK3:p-MLKL hetero-oligomer that functions as the protomer for the cytosolic formation of higher order p-RIPK3:p-MLKL oligomers (Fig. 6). In contrast to our hypothesis, previous work by Sun et al.^19^ showed that inhibition of MLKL did not interfere with the formation of RIPK3 dots. However, we point out that Sun et al.^19^ showed the effect of NSA treatment on the distribution of GFP-RIPK3 following MLKL knockdown in HeLa cells. Our results demonstrate that NSA is autofluorescent and leaks in the FITC (green) channel (Supplementary Fig. 11), and so it should not be included in fluorescence microscopy based on green fluorescence. Similar issues in the results reported by Sun et al. may have contributed to differences in data interpretation.

According to current knowledge of necroptotic signaling, the pseudokinase MLKL is activated by RIPK3-dependent phosphorylation of its pseudokinase domain, which causes a conformational change that exposes the membrane-permeabilizing 4HBD^9^. We observed that MLKL was also phosphorylated in the nucleus, suggesting that its phosphorylation occurs upstream of cytosolic necrosome formation (Fig. 4). However, phosphorylation and oligomerization of MLKL in the nucleus apparently did not lead to execution of its effector function (membrane permeabilization) because the nuclear envelope was apparently not disintegrated (Fig. 5a). In this respect, nuclear phosphorylated MLKL could fulfill a canonical pseudokinase function by acting as an allosteric activator of RIPK3 in order to promote correct protomer formation^44^.

The subsequent cytosolic assembly of phospho-RIPK3:phospho-MLKL protomers into higher order signaling complexes would increase the local concentration of phospho-MLKL, possibly allowing proximity-driven conformational rearrangements and/or additional phosphorylation of MLKL, which have been shown to tune MLKL activation^44^. Consequently, MLKL would be further activated on the scaffold of cytosolic phospho-RIPK3:phospho-MLKL oligomers, resulting in MLKL translocation and recruitment to the plasma membrane and consequent membrane permeabilization^6–10, 45^.

There is a recent description of the accumulation of p-MLKL, active RIPK3 and active RIPK1 in the nucleus before the onset of necroptosis or induction of the NLRP3 inflammasome^21^. Considering that MLKL can cell-intrinsically trigger the NLRP3 inflammasome^46^, those findings could point to an early nuclear checkpoint where the decision between inflammation and necroptosis induction is made. By using nuclear import and export inhibitors combined with genetic approaches, we identified a nuclear checkpoint, further downstream in the necroptotic signaling pathway, which controls the formation of the cytosolic necrosome and consequently induction of cell death. Moreover, the observations of Yoon et al.^21^ and our study show that the necroptotic signaling pathway does not represent a simple complex I to II transition in the cytosol, but involves compartmentalization of key necroptotic players into the nucleus.

As a platform, RIPK3 sits in control of both necroptotic and apoptotic complexes^15–17, 24^, whereas active RIPK3 kinase is required for necroptosis but not apoptosis^47, 48^. Our results provide evidence that RIPK3 kinase is active in the nucleus during necroptosis, implying that a RIPK3-activating complex is assembled in the nucleus (Figs. 2, 4). This compartmentalization in the nucleus provides a spatial separation from apoptotic signaling complexes, which are formed in the cytosol^2^. Thus, it may not be the level of respective downstream effectors that determines an apoptotic or necroptotic signaling outcome, as suggested by Cook et al.^15^, but rather the intracellular localization of RIPK3-containing death complexes. Although many questions remain about this nuclear checkpoint in necroptotic signaling, the compartmentalization of key necroptotic signaling events in the nucleus could be a mechanism that ensures correct interpretation of necroptotic vs. apoptotic death signals.

## Methods

### Constructs

The sequences encoding wild-type human RIPK1, human RIPK3, and human MLKL were cloned in pENTR3C using the CloneEZ PCR Cloning Kit (GenScript). The NLS sequence of SV40 large T was N-terminally fused to the coding sequence. Next, these sequences were transferred into homemade, modified pCDNA 3.1 vector backbones (vector map, Supplementary Fig. 12) using the LR Gateway recombination system (Life Technologies) resulting in N-terminally tagged RIPK3 with flag (FLAG-RIPK3) or EGFP-RIPK3 (GFP-RIPK3), and C-terminally tagged MLKL with EGFP (MLKL-EGFP). For mCherry-RIPK3, the *EGFP* gene of pDest-EGFP-hRIPK3FL was replaced by the mCherry cassette of pmCherry-C1 (Clontech) using the CloneEZ PCR Cloning Kit (GenScript). RIPK3 RHIM (Addgene ID 41385) and D160N (Addgene ID 41386) were purchased from Addgene and the coding sequence was cloned in pENTR3C. RIPK3 K50A was generated by QuickChange mutagenesis (Agilent Genomics) of GFP-NLS-RIPK3 and GFP-RIPK3. MLKL-GFP (T357A/S358A) and MLKL-GFP (T357E/S358D) were generated by by QuickChange mutagenesis (Agilent Genomics) of MLKL—GFP. All generated plasmids have been deposited at the plasmid collection of the Belgian Coordinated Collections of Microorganisms (BCCM) (http://bccm.belspo.be/catalogues/lmbp-plasmids-catalogue-search).

### Cell lines and treatments

FADD-deficient Jurkat T cells (purchased from ATCC) were cultured in Roswell Park Memorial Institute (RPMI). HeLa cells (ECACC) and SV40 large T-immortalized MEF cells (generated in house) were cultured in Dulbecco’s modified eagle’s minimal essential medium. Both media were supplemented with 10% fetal calf serum (FCS) and l-glutamine (200 mM). Cell cultures were routinely tested for mycoplasma contamination. HeLa cells were transfected using JetPrime reagent (Polyplus transfection) according to manufacturer’s instructions. After 24 h, the cells were pre-treated for 30 min with 20 μM z-Val-Ala-DL-Asp(Ome)-fluoromethylketone (zVAD-fmk) (Bachem AG), and 2 μM BV6 (Selleck) or 10 μM TGF-*β*-activated kinase 1 inhibitor (TAKi) (AnalytiCon Discovery GmbH), followed by treatment with human TNF (600 lU/ml). Ten micromolar Necrostatin-1 (Nec-1) (Calbiochem, Merck) and 10 μM Necrosulfonamide (NSA) (Toronto Research chemicals) were included in the pre-treatment. Leptomycin B (LMB) (Sigma-Aldrich) was used at a concentration of 1 μM for Hela cells and 0.25 μM for FADD-deficient Jurkat cells and was added at the time of transfection or 1 h before TNF treatment or confocal imaging. GppNHp (non-hydrolyzable GTP analog, Sigma-Aldrich) was added at 1 mM 2 h before treatment or confocal imaging.

### Subcellular fractionation

Nucleus-enriched fractions of FADD-deficient Jurkat cells were obtained as described^49^. Briefly, cells were swollen in hypotonic buffer and ruptured by dounce homogenization. After clearing lysates at 100×*g* for 5 min, nucleus-enriched fractions were pelleted at 1300×*g* for 10 min. Cytosolic fractions were obtained by centrifugation of the supernatants at 17,000×*g* for 10 min. Nuclear extracts of HeLa cells were obtained as described^50^. Briefly, cells were swollen in hypotonic buffer, followed by fractional lysis in NP-40. After differential spinning, nucleus-enriched fractions and cytosolic fractions were collected. To compare posttranslational modifications of cytosolic and nuclear RIPK1 and MLKL, protein levels were equalized between nucleus-enriched and cytosolic fractions by loading four times more of the nucleus-enriched fraction than the cytosolic fraction.

### Cell death measurement

HeLa cells transfected with GFP-fused constructs were collected and left to recover before staining with SYTOX Blue (Life Technologies) at a final concentration of 5 μM. Cell death in the GFP-positive cell population was determined by analyzing SYTOX Blue positive cells on a FACSVerse flow cytometer (BD Biosciences) and by using FlowJo software (gating strategy see Supplementary Fig. 13). Jurkat cells were stained with 5 μM SYTOX Green (Life Technologies) and analyzed by using a FLUOstar Omega fluorescence plate reader (BMG Labtech GmbH, Ortenberg, Germany). Percent cell death was calculated as follows:

$${\begin{array}{ccccc}\\ & \left[ {100 \times \left( {{\mathrm{induced}}\,{\mathrm{fluorescence - background}}\,{\mathrm{fluorescence}}} \right)} \right]\\ & {\mathrm{ \div }}\left( {{\mathrm{maximal}}\,{\mathrm{fluorescence}}\,{\mathrm{achieved}}\,{\mathrm{by}}\,{\mathrm{Triton X 100}}}\right.\\ & \left.{{\mathrm{permeabilization - background}}\,{\mathrm{fluorescence}}} \right).\\ \end{array}}$$

The data are presented as mean ± S.E.M of at least 2–3 independent experiments. All cell death profiles in this paper are shown as averages ± S.E.M. A comparison of groups was performed using paired, two-sided Student’s *t*-test assuming normal distribution. A value of *p* < 0.05 was considered to indicate statistical significance.

### Antibodies

The following antibodies were used for Western blotting or immunostainings: anti-FLAG-HRP (Sigma-Aldrich; Cat. No. A8592, Lot. No. 116K6031), anti-RIPK1 (BD Bioscience, Cat. No. 610459), anti-RIPK3 (Abcam, Cat. No. ab56164), anti-MLKL (Genetex, Cat. No. GTX107538, Lot. No. LN40030), anti-p-MLKL (Abcam; Cat. No. 187091; Lot No. GR12667-11), anti-Histone (Santa Cruz; Cat. No. sc-8030, Lot No. L0808), anti-LDHA (Cell Signaling; Cat. No. 2012, Lot No. LN002), anti-Flotillin (Cell Signaling; Cat. No. 3253 S, Lot No. 10/2013), anti-tubulin-HRP (Abcam; Cat. No. ab21058, Lot. GR260583-2). For immunostainings, the following antibodies were used: anti-p-hMLKL S358 (Abcam; Cat. No. ab187091; Lot No. GR12667-11); anti-p-mMLKL S345 (Abcam; Cat. No. ab196436; Lot No. GR246882-1); anti-p-hRIPK3 S227 (Abcam; Cat. No. ab209384; Lot No. GR257915-8); anti-p-mRIPK3 T231 + S232 (Abcam; Cat. No. ab205421; Lot No. GR268165-4) or S232 (Abcam; Cat. no 195117; Lot No. GR268165-4). Full scans of all Western blots presented in figures or Supplementary Figures are shown in Supplementary Figs. 14–18.

### Intracellular localization by confocal microscopy

HeLa cells were transfected with GFP-tagged RIPK3 or a combination of mCherry-RIPK3 and MLKL-GFP constructs using Jetprime transfection (Polyplus Transfection) according to manufacturer’s protocol. After 16 h, the cells were treated with TBZ and nuclei were counterstained with Hoechst. The cells were either fixed with 1% PFA or left unfixed. For immunostaining, transiently transfected HeLa cells were fixed with 1% PFA, permeabilized in 0.1% Triton, and blocked with 0.2% donkey serum in PBT buffer (PBS + 0.1% Tween 20). The cells were then incubated overnight with the indicated antibodies followed by incubation with DyeLight 568 conjugated secondary antibodies for 1 h. Nuclei were stained with Hoechst. Cells were imaged by confocal microscopy using an LSM780 or LSM880 confocal microscope (Zeiss) and the observer was blinded to the identities of specimens. Images (8 bit) were acquired with a Plan-Apochromat 63X/1.4 oil objective at a resolution of 1528 by 1528 pixels (pixel size 70 nm × 70 nm). The microscope is equipped with a Ti:Sa laser (MaiTai DeepSee, SpectraPhysics) to image Hoechst and an Ar laser to excite GFP. The scanhead contains 2 PMTs and a QUASAR detection unit. Images were processed using Fiji imaging software. For quantification, cells were imaged using a spinning disk confocal microscope (Zeiss) equipped with a Rolera EMCCD camera. A Plan-Apochromat 40X/1.4 Oil DIC (UV) VIS-IR M27 objective was used. Pixel sizes were 0.167 by 0.167 μm and *z*-step size 0.240 μm. EGFP was excited by a 488-nm diode laser and Hoechst by a 405-nm diode laser. Quantification of nuclear and cytosolic fractions was done using Volocity 3D Image Analysis Software (PerkinElmer, UK). By means of 3D segmentation, the volume of total protein and the volume of protein in the nucleus were determined for each cell to calculate the nuclear fraction of the protein in each cell. For each condition, 7–9 fields of view were randomly selected and imaged. Cells within these fields were analyzed, resulting in 12–60 data points per condition. All quantitative datasets in this publication are presented as aveages ± S.E.M.

### MLKL oligomerization

Formation of MLKL oligomers was previously analyzed by the cysteine crosslinker Bismaleimidohexane (BMH), which chemically joins two or more molecules by a covalent disulfide bond^45^. We analyzed the formation of MLKL oligomers by using the redox catalyst CuPhe (20 mM 1,10 phenanthroline with 300 mM CuSO_4_)^51^. Like BMH, copper (II)(1,10-phenanthroline)3 (CuPhe) induces the formation of disulfide bonds, but it is more efficient. The oxidation with CuPhe yielded disulfide-linked MLKL containing low-molecular weight complexes. Nucleus-enriched fractions isolated form control- and TNF-treated FADD-deficient Jurkat cells were incubated with CuPhe for 30 min on ice. The reaction was then quenched by incubation with 100 mM EDTA for 15 min. Oligomerization of MLKL was analyzed by SDS–PAGE under non-reducing and reducing conditions.

### Immunoprecipitation

Nucleus-enriched fractions were lysed in 6 M urea. Before immunoprecipitation using protein A-pre-coupled anti-ubiquitin Dynabeads (Invitrogen), lysates were diluted to 0.2 M urea with lysis buffer (20 mM Tris-HCl, pH 7.4, 135 mM NaCl, 1.5 mM MgCl_2_, 1 mM EGTA, 10% glycerol, 1% NP-40). After overnight incubation, beads were washed in lysis buffer and immunoprecipitates were eluted in Laemmli buffer (50 mM Tris-HCl (pH 6.8), 2% SDS, 10% glycerol). Immunoprecipitates were analyzed by immunoblotting.

### RNA interference

Control and MLKL siRNA ON-TARGET SMARTpools were obtained from Dharmacon. HeLa cells were transfected with the siRNAs using Interferin (Polyplus Transfection) according to manufacturer’s instructions.

### Data availability

All the relevant data that are not in the article or Supplementary Files are available from the authors upon request. Plasmid constructs generated for this study have been deposited at the plasmid collection of the Belgian Coordinated Collections of Microorganisms (BCCM). LMPB codes or Addgene ID’s are provided in Supplementary Data 1.

## Electronic supplementary material

Supplementary Information

Description of Additional Supplementary Files

Supplementary Data 1
