# Supplementary material for: Nuclear RIPK3 and MLKL contribute to cytosolic necrosome formation and necroptosis
Source: Commun Biol. 2018 Jan 22;1:6. doi: 10.1038/s42003-017-0007-1 (PMC6123744; doi:10.1038/s42003-017-0007-1)
Supplement: Supplementary file 1 — Supplementary Information [file 42003_2017_7_MOESM1_ESM.pdf]

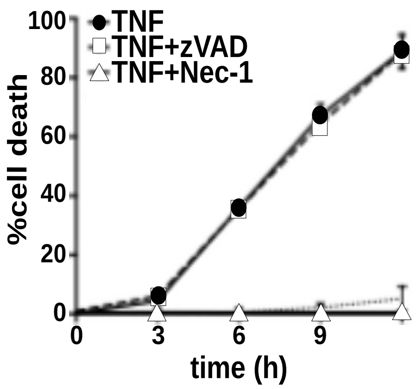

Supplementary Figure 1. TNF induces necroptosis in FADD-deficient Jurkat cells

Cell death profile of FADD-deficient Jurkat cells pre-treated with zVAD or Nec-1 followed by TNF treatment for indicated times by analyzing SYTOX Green uptake. n=5.

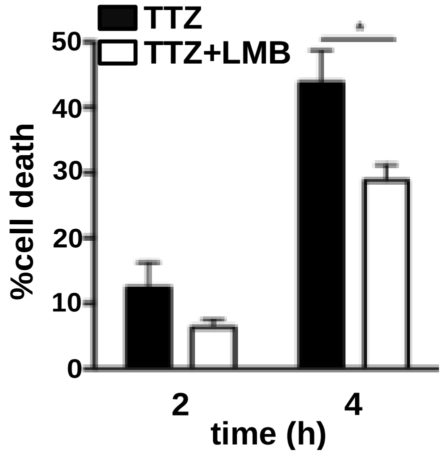

Supplementary Figure 2. LMB reduces TTZ-induced necroptosis in MEF cells

Cell death profile of MEF cells treated TNF, TAKi and zVAD (TTZ) in combination with or without LMB at indicated hours by analyzing SYTOX Green uptake. n=3; **P*<0.01

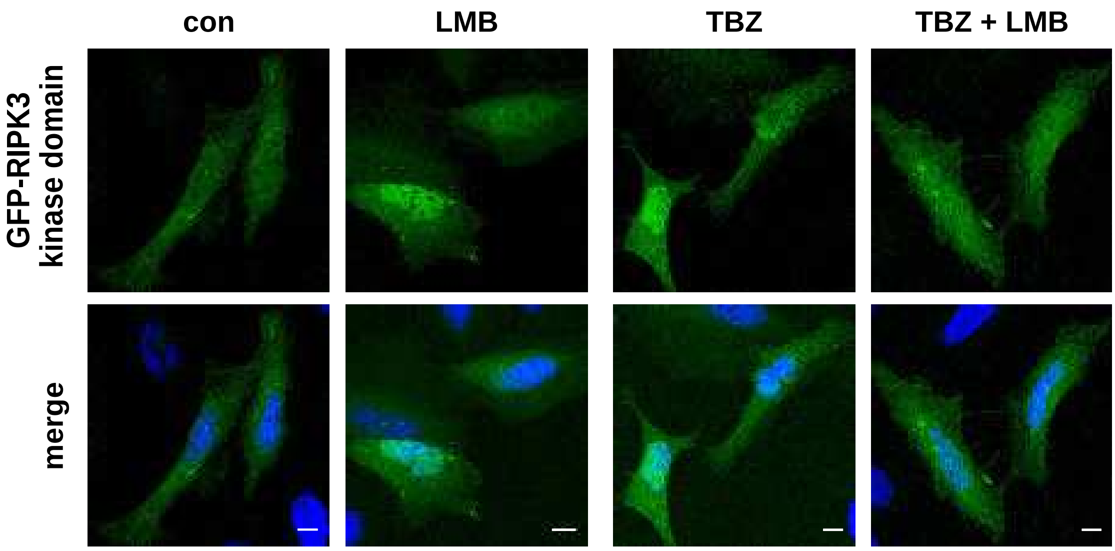

Supplementary Figure 3. Kinase domain of RIPK3 mediates nucleo-cytoplasmic shuttling

Confocal images of single optical sections of HeLa cells transiently expressing GFP-RIPK3 kinase domain (aa 1-292) treated with -/+ LMB and control (con) or TBZ. Scale bars: 10µm.

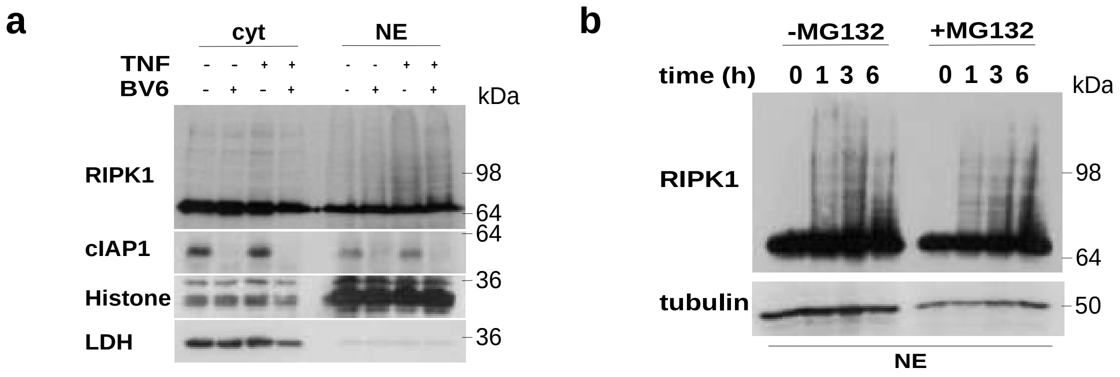

Supplementary Figure 4. Non-degradative, nuclear RIPK1 ubiquitination is not mediated by cIAPs

(a) Immunoblot of RIPK1 in cytosolic and nuclear enriched fractions of FADD-deficient Jurkat cells treated with BV6 or control (con) and TNF for 3 h. **(b)** Immunoblot of RIPK1 in cytosolic and nuclear enriched fractions of FADD-deficient Jurkat cells treated with MG132 or control (con) and TNF for 3 h. All immunoblots are representative of two independent experiments. Uncropped images of immunoblots are shown in Supplementary Figure 16.

.

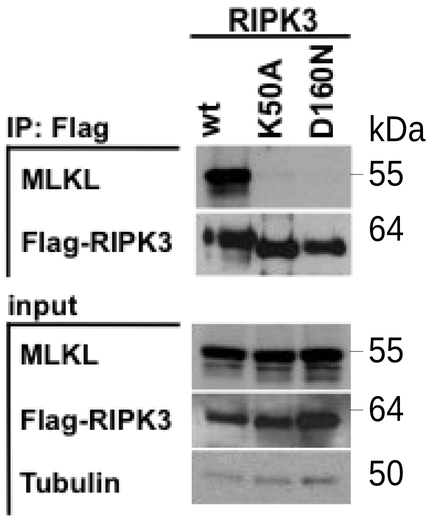

Supplementary Figure 5. Kinase dead RIPK3 does not interact with MLKL

Immunoblot of MLKL on Flag-RIPK3 immunoprecipitations of HeLa cells expressing Flag-wtRIPK3, Flag-RIPK3 K50A or Flag-RIPK3 D160N. Immunoblots are representative of two independent experiments. Uncropped images of immunoblots are shown in Supplementary Figure 16.

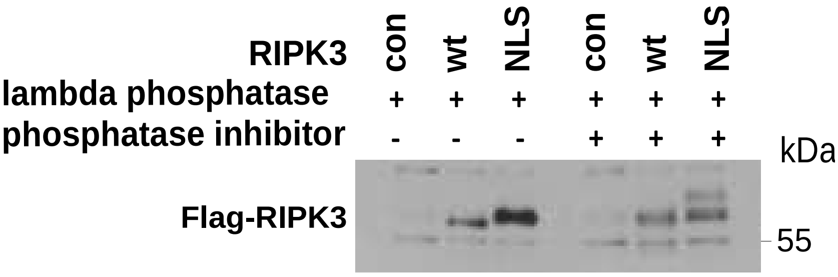

Supplementary Figure 6. RIPK3 is phoshorylated in the nucleus

Immunoblot of Flag on nuclear enriched fractions of Flag-RIPK3 or Flag-NLS-RIPK3 expressing HeLa cells treated with TBZ subjected to Lambda phosphatase or Lambda phosphatase+phosphatase inhibitor treatment. Immunoblot is representative of two independent experiments. Uncropped images of immunoblots are shown in Supplementary Figure 16.

.

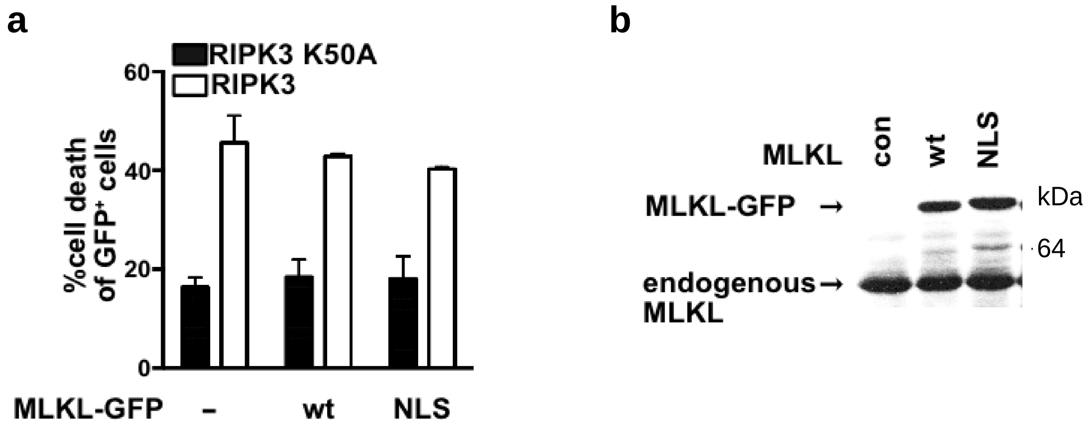

Supplemental Figure 7. Overexpression of MLKL or MLKL-NLS does not confer increased sensitivity to necroptosis in HeLa cells

(a) Cell death profile of HeLa cells transiently transfected with flag-RIPK3 or the kinase dead RIPK3 mutant K50A and MLKL-GFP or MLKL-NLS-GFP analyzing SYTOX Blue uptake in the GFP+ population. n=3. **(b)** Immunoblot of MLKL of HeLa cells transiently transfected with Flag-RIPK3 (con) or together with MLKL-GFP or MLKL-NLS-GFP. Immunoblot is representative of two independent experiments. Uncropped images of immunoblots are shown in Supplementary Figure 16.

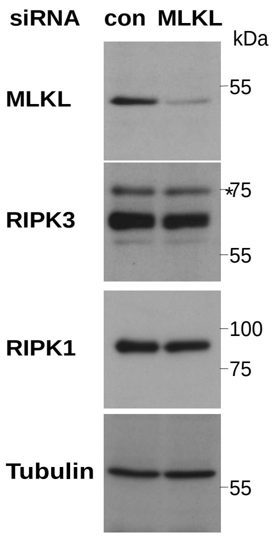

Supplemental Figure 8. RIPK3 expression levels in control or MLKL siRNA transfected HeLa cells

Immunoblots of MLKL, RIPK3 and RIPK1 from Flag-RIPK3 expressing HeLa cells transfected with control (con) siRNA or MLKL siRNA. Asteriks denotes unspecific band. All immunoblots are respresentative of two independent experiments. Uncropped images of immunoblots are shown in Supplementary Figure 18.

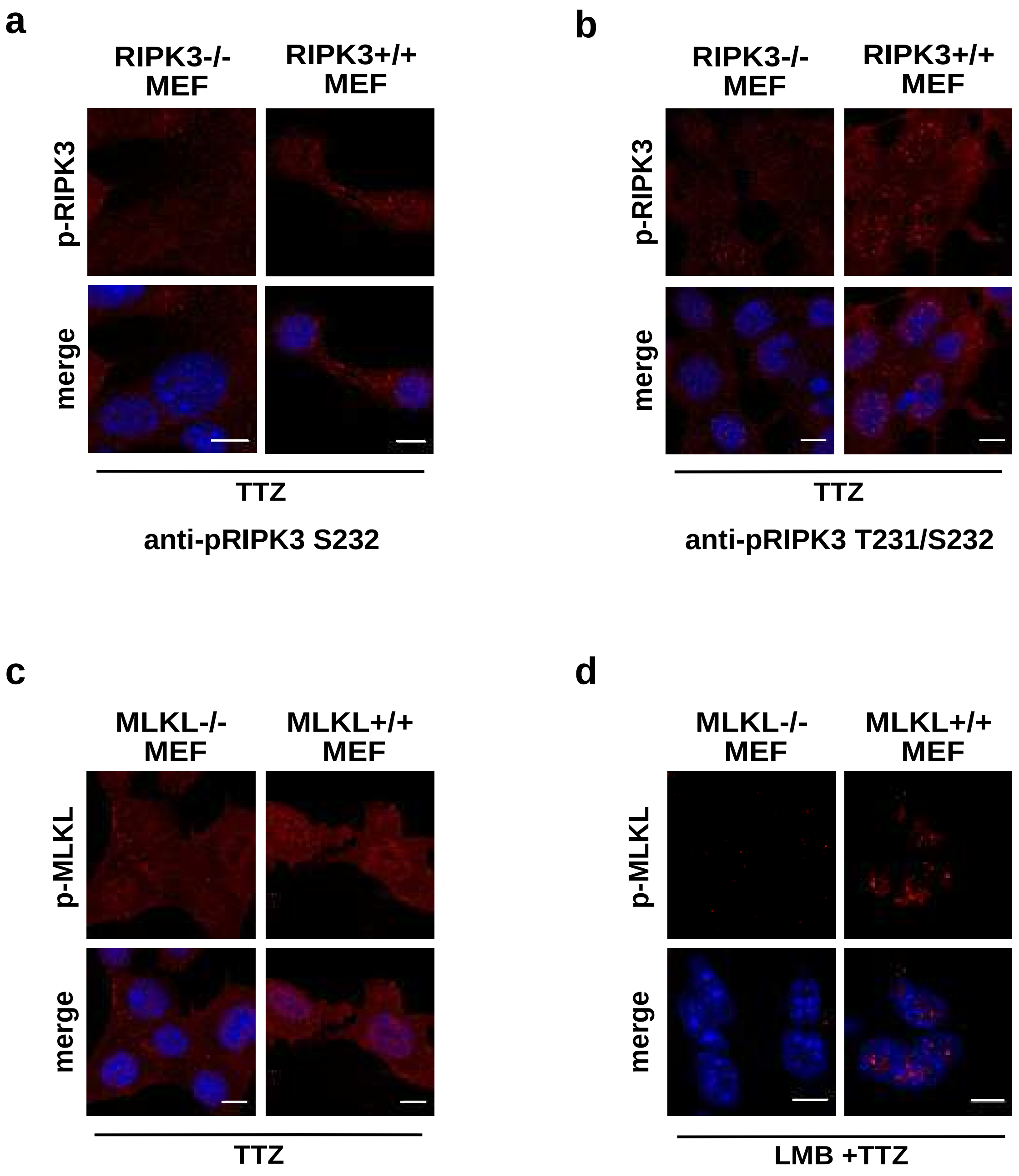

Supplementary Figure 9. Mouse anti-pRIPK3 and mouse anti-pMLKL antibody tryout.

(a-d) Confocal images of single optical sections of **(a, b)** Ripk3-/- and Ripk3+/+ MEF cells treated with TTZ for 2 h and immunostained with **(a)** anti-p-RIPK3 S232 (Abcam; Cat. no 195117) or **(b)** anti-p-RIPK3 T231/S232 (Abcam; Cat. no 205421) **(c,d)** Mlkl-/- and Mlkl+/+ Mef cells immunostained with anti-p-MLKL antibody S345 (Abcam, Cat. No 196436) and treated with **(c)** TTZ or **(d)** TTZ+LMB. Scale bars for all panels: 10μm.

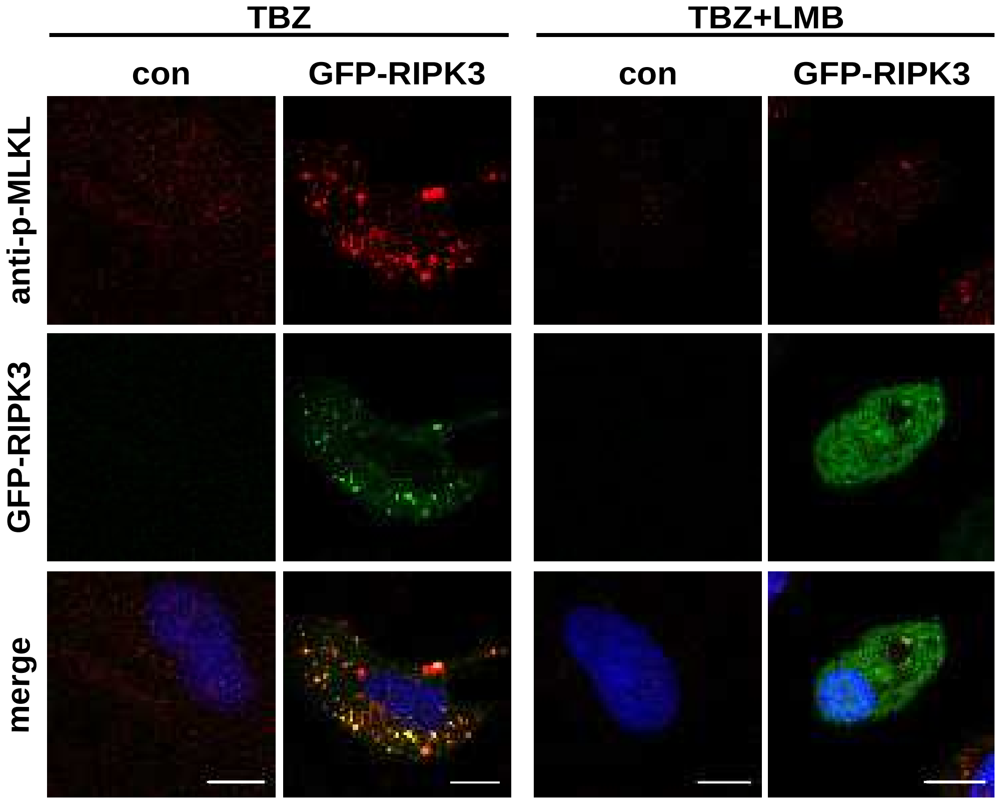

Supplementary Figure 10. LMB prevent cytosolic p-MLKL punctae immunofluorescence

Confocal images of single optical sections of HeLa cells transiently expressing control vector or GFP-RIPK3 treated with left two panels TBZ or right two panels TBZ+LMB followed by anti-p-MLKL immunostaining. Scale bars: 10μm.

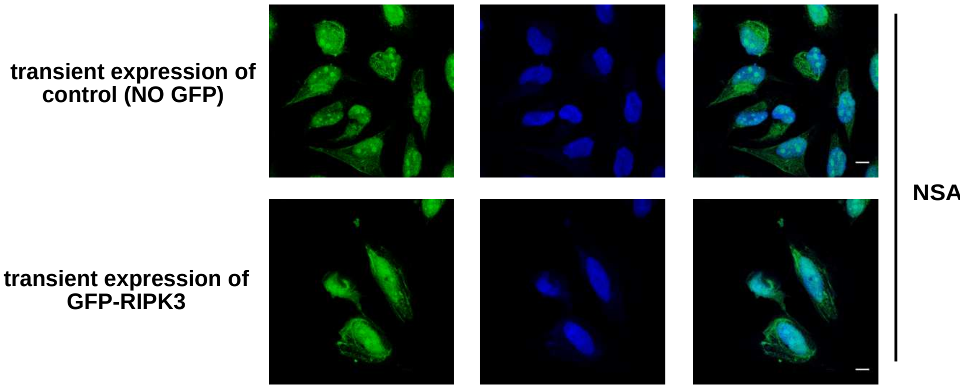

Supplementary Figure 11. NSA is autofluorescent

Confocal imaging of single optical sections of HeLa cells transiently transfected with control vector (no GFP) or with GFP-RIPK3 and treated with 10 μM NSA. Images are representative of two independent experiments. Scale bars, 10 μm.

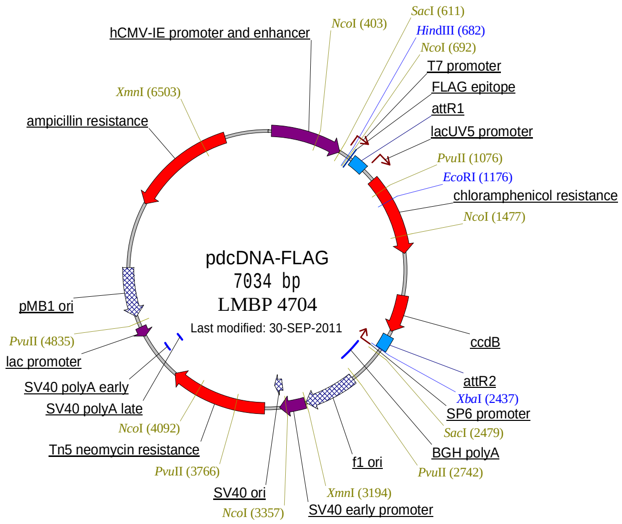

Supplementary Figure 12. Vector map of the homemade, modified pCDNA 3.1 vector backbone

The plasmid was constructed by converting pcDNA3 to a Gateway destination vector as follows: 1) The Gateway reading frame cassette A (rfA, from Invitrogen) was amplified with primers containing a HindIII site, the FLAG tag and an XbaI site. 2) The PCR product and the vector pcDNA3 (Invitrogen) were digested with HindIII and XbaI and ligated together, resulting in pdcDNA-FLAG. pdcDNA-FLAG is a Gateway destination vector, containing the ccdB gene flanked by the bacteriophage λ attR recombination sites. pdcDNA-FLAG is designed for fusing a gene of interest to the N-terminal FLAG epitope tag according to the Gateway Cloning Technology (Invitrogen) and for high-level, constitutive, native expression of this gene in mammalian cells under control of the human CMV-IE promoter and enhancer.

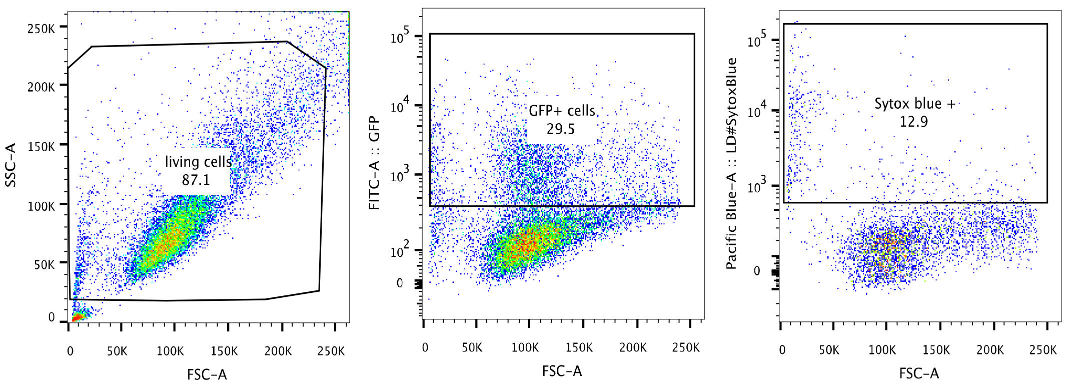

Supplementary Figure 13. Gating strategy to analysing cell death in GFP+ cell population

GFP-RIPK3 transfected HeLa cells were stained with Sytox blue and analyzed by flow cytometry. Cell debris were initially gated away using the forward scatter (FSC) and sideward scatter (SSC). First gate was sub-gated for GFP+ cells using FITC and FSC. GFP+ cells were further sub-gated for Sytox blue+ cells using Pacific Blue and FSC.

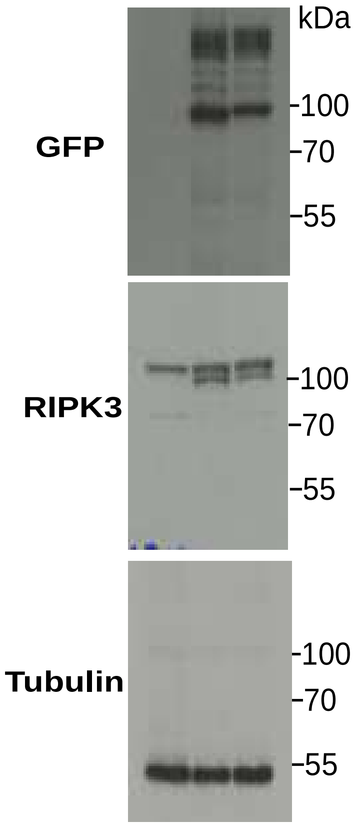

Supplementary Figure 14. Full-sized scans of Immunoblots in Figure 1e.

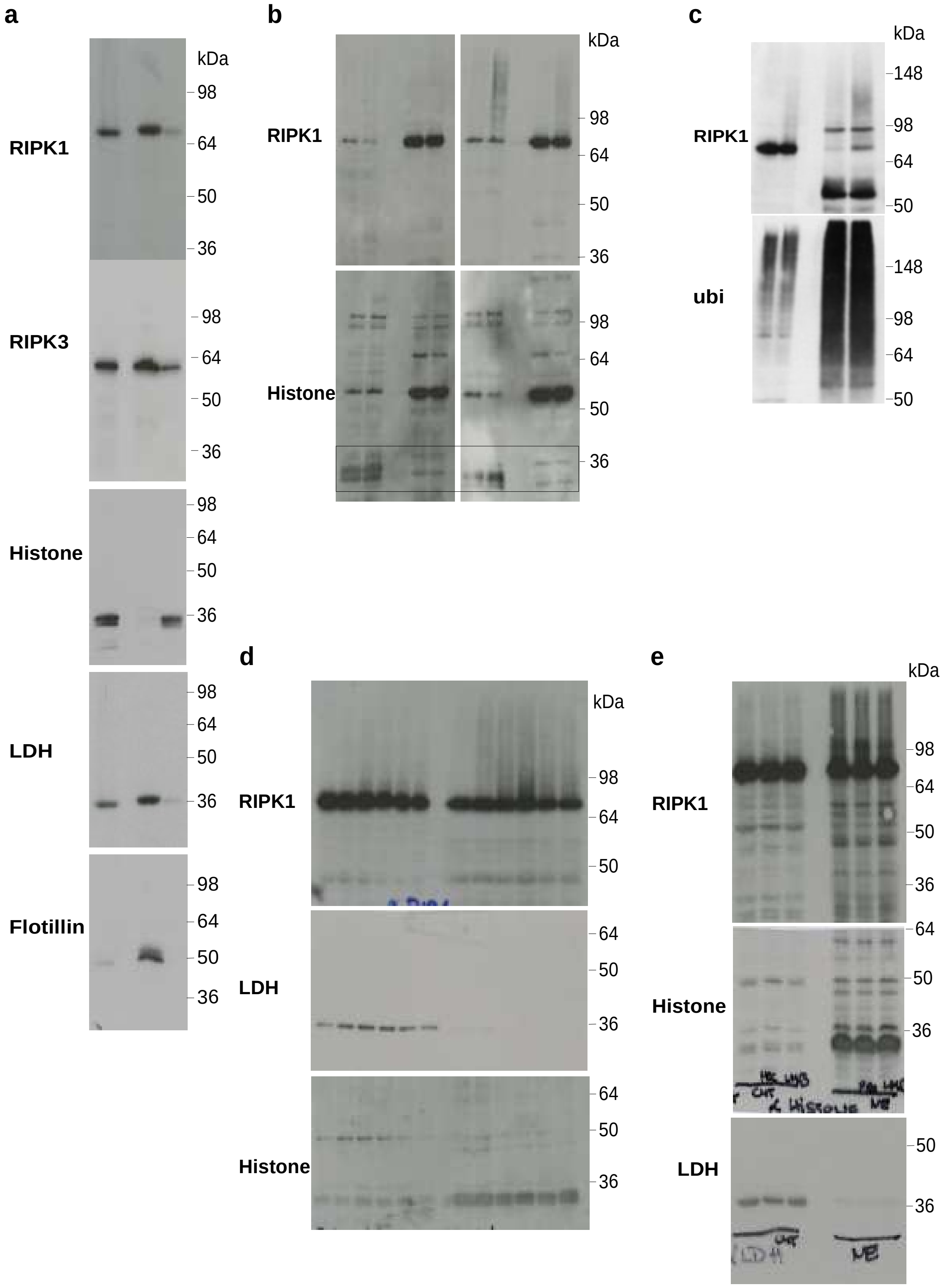

Supplementary Figure 15. Full-sized scans of Immunoblots in Figure 2

a. Figure 2c; b. Figure 2e; c. Figure 2f; d. Figure 2g; e. Figure 2h.

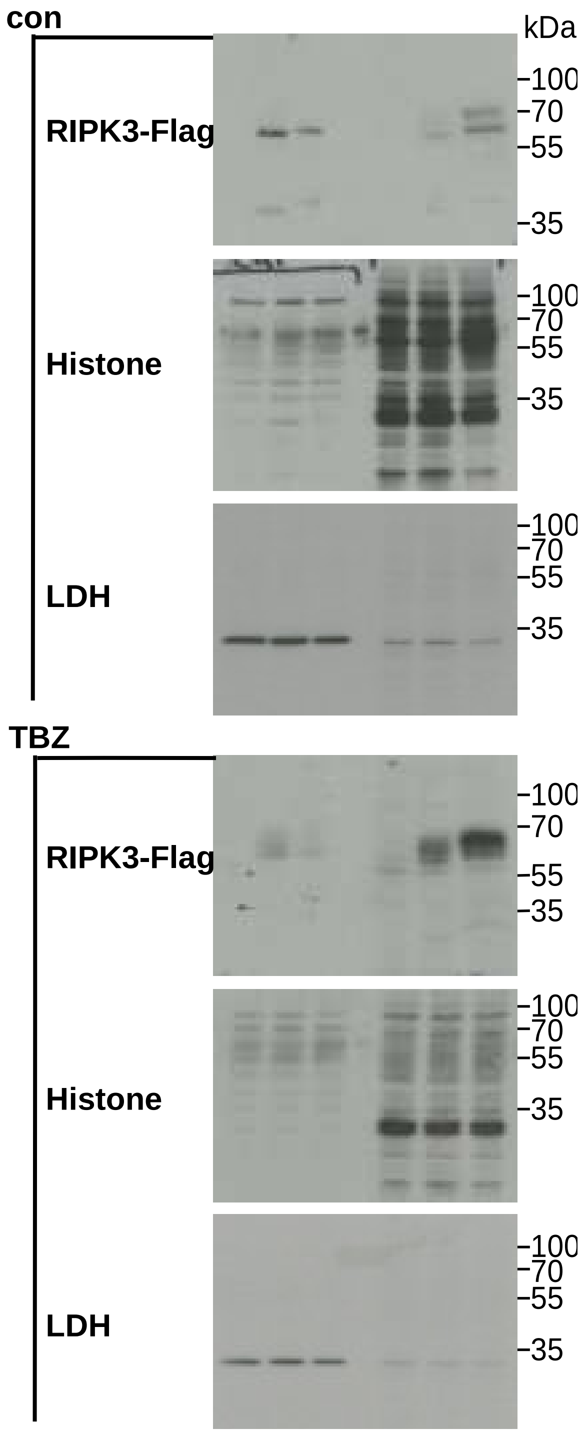

Supplementary Figure 16. Full-sized scans of Immunoblots in Figure 3d.

a

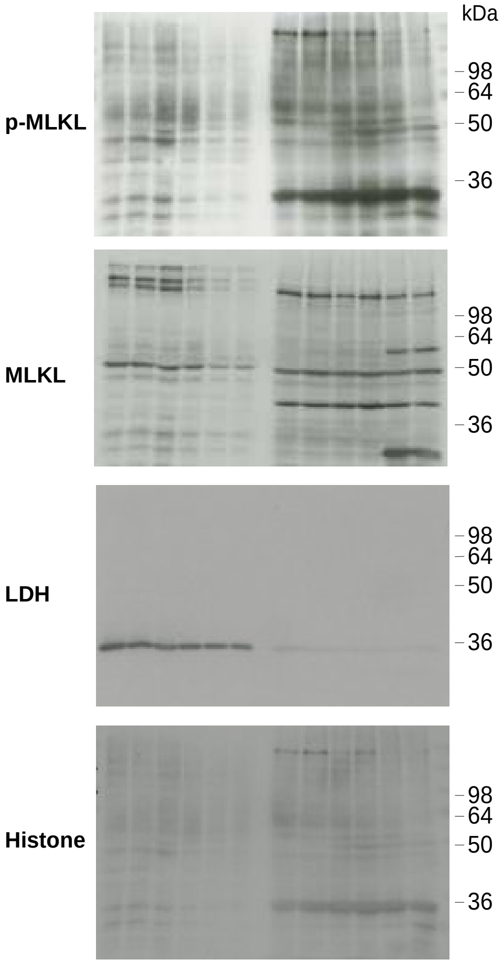

b

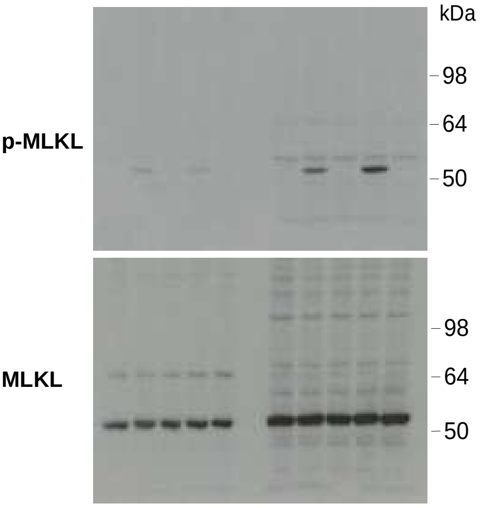

Supplementary Figure 17. Full-sized scans of Immunoblots in Figure 4

a. Figure 4a; b. Figure 4b.

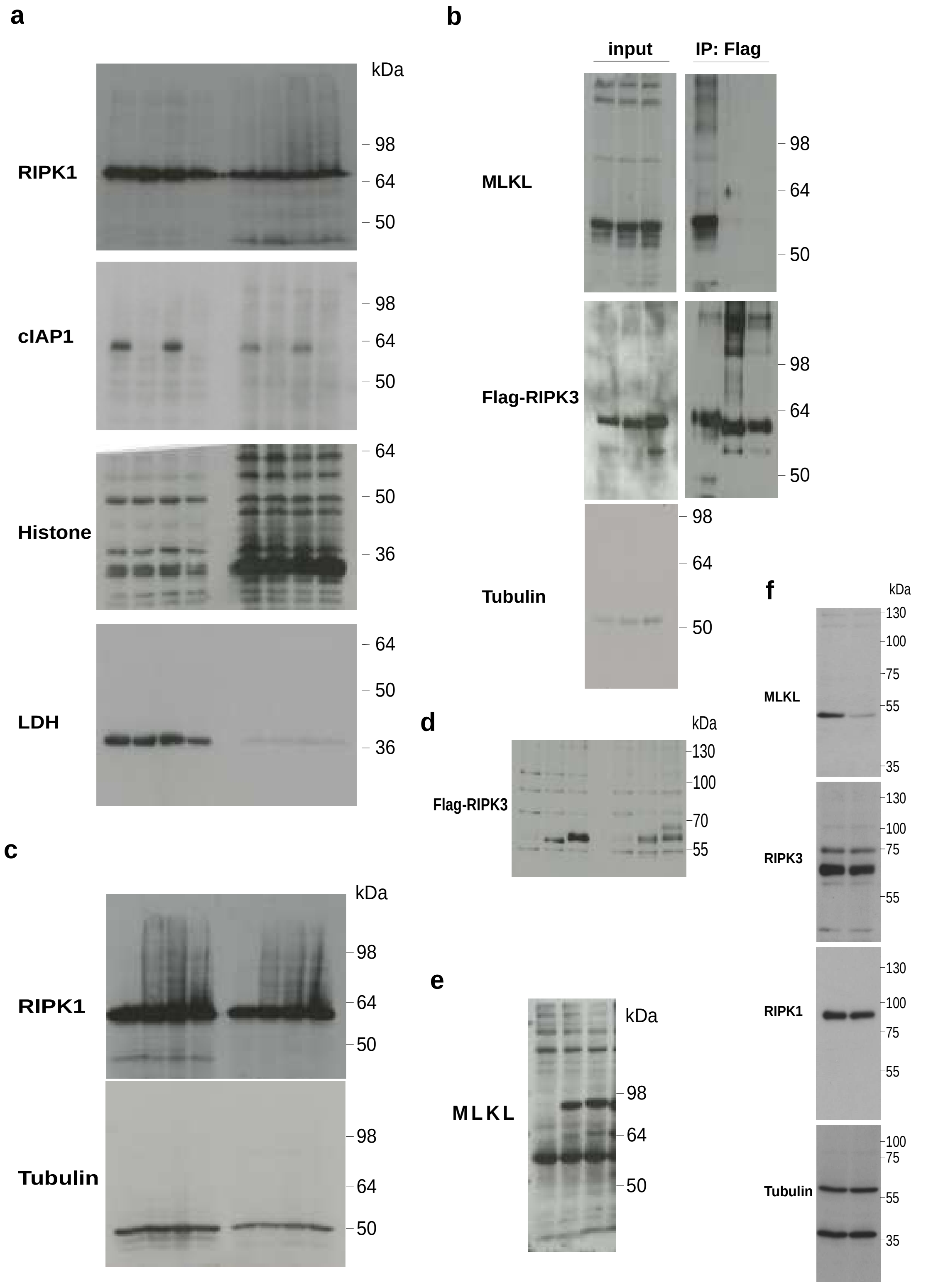

Supplementary Figure 18. **Full-sized scans of Immunoblots in Supplementary Figures 1-8**

a. Supplementary Fig. 4a; b. Supplementary Fig. 4b; c. Supplementary Fig. 5; d. Supplementary Fig. 6; e. Supplementary Fig. 7b; f. Supplementary Fig. 8.
