# Supplementary material for: Nuclear RIPK3 and MLKL contribute to cytosolic necrosome formation and necroptosis
Source: Commun Biol. 2018 Jan 22;1:6. doi: 10.1038/s42003-017-0007-1 (PMC6123744; doi:10.1038/s42003-017-0007-1)
Supplement: Supplementary file 2 — Description of Additional Supplementary Files [file 42003_2017_7_MOESM2_ESM.docx]

**Description of Additional Supplementary Files**

File Name: Supplementary Data 1

Description: List of newly-generated plasmids and database identity codes
